# Free Energy Perturbation Simulations Measure the Change in Binding Affinity of the Aβ25–35 Peptide to the Zwitterionic Bilayer Caused by Oxidation

**DOI:** 10.1021/acs.jcim.5c02148

**Published:** 2025-10-22

**Authors:** Xingyu Luo, Elias Khayat, Steven R. Bowers, Bryan M. Delfing, Christopher Lockhart, Dmitri K. Klimov

**Affiliations:** School of Systems Biology, 3298 George Mason University, Manassas, Virginia 20110, United States

## Abstract

We designed and employed a relative free energy perturbation (FEP) combined with replica exchange with solute tempering (REST) all-atom molecular dynamics to investigate how Met35 oxidation affects the free energy of binding of the Aβ25–35 peptide to the DMPC lipid bilayer. We first showed that our restraint-free FEP/REST protocol delivers a converged sampling of alchemical transformations in the DMPC bilayer and in lipid-free water. Then, we determined that Met35 oxidation moderately reduced the peptide binding free energy by ΔΔ*G*_b_ = 3.2 kcal/mol. Its reduction is driven by a partial cancellation of two large opposing factors. Oxidation makes binding less enthalpically favorable, but it also mitigates entropic losses. Ultimately, the entropic gain is insufficient to compensate for the enthalpic binding loss. Our analysis identified two sources of these energetic changes: (i) Met35 oxidation introduces minimal energetic frustration in water compared to the bilayer, and (ii) it produces strong entropic gains within the bilayer system. The latter takes place because Met35 oxidation disrupts the helical structure in Aβ25–35, expels the peptide from the bilayer core, and alleviates lipid disorder. Energetic and structural effects collected by us illuminate the molecular mechanism by which oxidation modulates Aβ25–35 properties, potentially explaining its reduced cytotoxicity.

## Graphical abstract

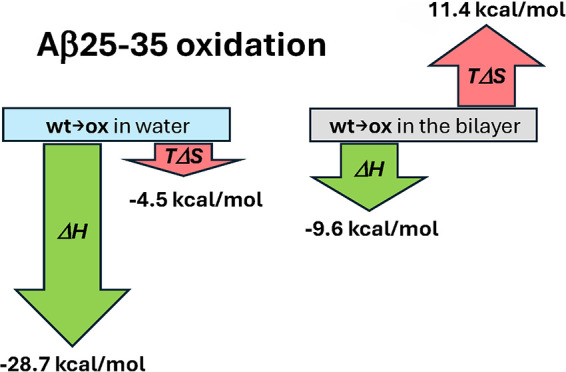

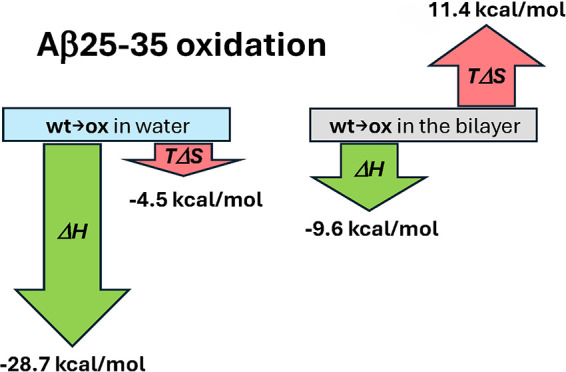

## Introduction

Computing the free energy Δ*G*_b_ of peptide ligand binding to a protein, DNA, or a lipid bilayer is a critical step in medicinal chemistry and the design of drugs.^1^ Over the years, different approaches have emerged to address this challenge. To perform virtual screening of ligands and evaluate Δ*G*_b_, one may use approximate scoring functions or employ artificial intelligence.^2,3^ However, this approach relies on training data for similar compounds and therefore may not be readily extendable to novel molecules. An alternative approach, which does not exploit ligand similarity, is based on computing molecular mechanics energy and implicit treatment of solvation using Poisson–Boltzmann and Generalized Born approximations.^4^ The method is typically applied *post priori* to the simulations of molecular complexes and their components. However, it suffers from difficulties in accurate accounting of entropic contribution, neglect of intermediate binding states, and errors caused by implicit treatment of water.^5^ A rigorous but computationally intensive approach for computing binding free energy is a free energy perturbation (FEP) method rooted in the Zwanzig formalism.^6,7^ FEP uses all-atom explicit solvent molecular dynamics simulations and is currently the most accurate method among in silico evaluations of binding affinities. FEP can estimate the absolute free energy of binding, Δ*G*_b_, or its relative changes, ΔΔ*G*_b_, associated with alchemical transformations.^7,8^ Both computations, however, require the development of thermodynamic cycles linking alchemical transformations with the free energy changes of interest.

If alchemical transformation involves few heavy atoms in a ligand and does not affect its binding pose or trapped water molecules, then molecular dynamics trajectories probing this transformation without enhanced sampling techniques might be sufficient for accurate evaluation of ΔΔ*G*_b_.^7^ However, if a ligand alternates between binding poses or the target molecule changes its conformation, standard molecular simulations would, in all likelihood, fail to converge.^9–13^ The sampling problem becomes even more acute if a ligand binds diffusively.^14^ Thereby, in all these cases traditional molecular simulations will not sample thermodynamically relevant conformational states and will produce limited or nonexistent overlap between the states from adjacent alchemical windows.^11^ The convergence problem can be mitigated by combining FEP with enhanced conformational sampling methods such as replica exchange. For instance, FEP + platform utilizes replica exchange with solute tempering (REST) to improve sampling of binding poses.^15^ A particular advantage of FEP/REST is computational efficiency stemming from targeted tempering of ligand regions involved in alchemical transformation.^15–17^ Recent studies by Jiang et al. have ported REST to the NAMD molecular dynamics program and demonstrated successful applications of FEP/REST by computing absolute free energies of *p*-xylene and *n*-butylbenzene binding to the L99A mutant of T4 lysozyme.^18,19^ These investigations were further expanded to probe the role of solvent in binding.^20,21^ Because FEP/REST simulations involve multiple replicas, they are computationally more demanding than traditional FEP, which does not consider parallel replicates of the simulation system. In return, FEP/REST may reduce errors in ΔΔ*G*_b_ down to 1 kcal/mol.^22^ However, recent reviews have pointed out that even FEP/REST may fail due to pronounced hysteresis along alchemical transformation.^13,23^

Recently, we have applied FEP/REST to evaluate the changes in binding free energies of the ligands binding diffusively to importin-α protein.^24^ Specifically, we showed that it is feasible to apply FEP/REST to compute binding energetics of the ligands forming no defined poses and capture variations in ΔΔ*G*_b_ down to ≲0.5 kcal/mol. However, it remains unclear whether the relative FEP/REST methodology can be extended to more complex problems, such as the binding of peptides to lipid bilayers. Up to now this case has been rarely studied^25^ and is challenging for several reasons. First, peptides bound to the bilayer are not expected to adopt a single conformation or pose within the bilayer. Furthermore, upon alchemical transformation, a peptide may adopt new conformations and/or binding poses. Finally, the lipid bilayer structure may also adjust in response to alchemical transformation. These challenges warrant the combination of FEP with enhanced sampling. The case study, in which we attempt to address them, is the evaluation of changes in binding affinity of Aβ25–35 peptide to dimyristoylphosphatidylcholine (DMPC) bilayer caused by oxidation.

Amyloid-β (Aβ) peptide is a natural product of cellular proteolysis of membrane-spanning β-amyloid precursor protein. Although Aβ peptides are involved in Alzheimer’s Disease (AD), their specific role remains elusive. An amyloid cascade hypothesis stating that Aβ amyloid fibrils are the causative agents in AD^26^ has been recently replaced with the realization that Aβ oligomers are cytotoxic and are better correlated with AD progression.^27–29^ Experimental characterization of Aβ peptides proved to be difficult due to their metastability and polymorphism.^30^ Furthermore, post-translational modifications of Aβ peptides further increase variance in their physicochemical properties.^31,32^ Nevertheless, one of the primary Aβ cytotoxic mechanisms is believed to be their interaction with neuronal membranes.^33^ As a candidate for our study, we selected an 11-mer Aβ fragment, Aβ25–35, shown in Figure 1a, which retains amyloidogenic and cytotoxic propensities of a parent full-length Aβ.^34–37^ Aβ25–35 spans a polar extracellular N-terminus (25–28) and a hydrophobic membrane embedded in a C-terminus (29–35) (Figure 1a). Importantly, Aβ25–35 is a naturally occurring peptide resulting from proteolysis.^38,39^ Experimental studies indicated that the Aβ25–35 propensity for aggregation and cytotoxicity may even eclipse those of full-length Aβ.^40,41^

**Figure 1:**
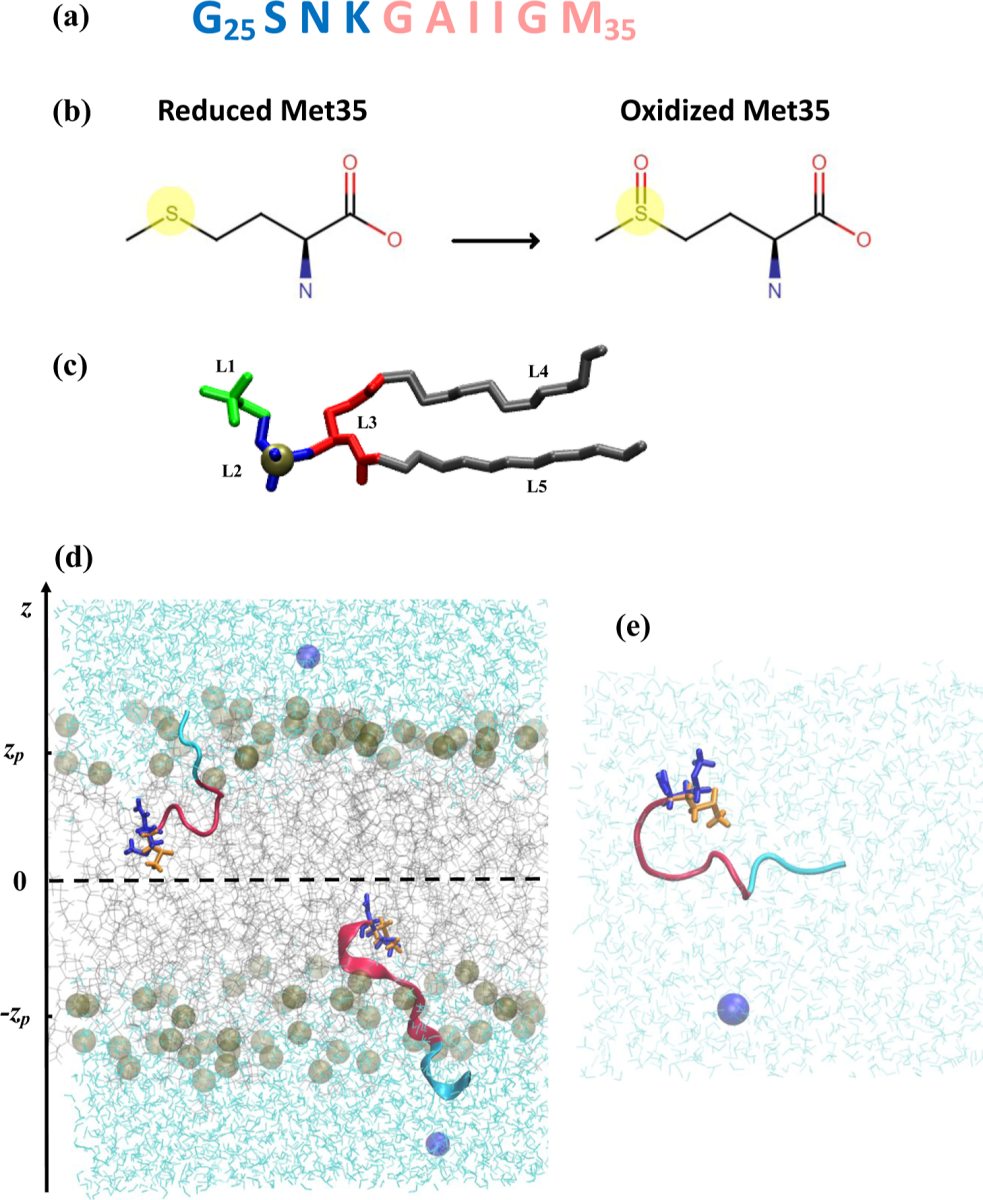
(a) Sequence of Aβ25–35 peptide. Polar N-terminal and hydrophobic C-terminal regions are in blue and red. (b) Oxidized Met35 side chain contains an oxygen atom, making this amino acid polar. (c) DMPC lipid contains five structural groups: Choline (L1), phosphate (L2) with phosphorus P atom in tan, glycerol backbone (L3), and two fatty acid tails (L4 and L5). L1-L3 constitute polar headgroups, whereas L4 and L5 make up the hydrophobic core. (d-e) Snapshots of simulation systems undergoing wt → ox alchemical transformation at (*T*_9_ = 440 *K*, λ_9_ = 0.5). In (d), Aβ25–35 peptides bind the DMPC bilayer. In (e), Aβ25–35 is placed in lipid-free water. Lipids are in gray, whereas the peptide is in cartoon representation. Reduced and oxidized Met side chains simultaneously present in the system are colored in blue and orange and shown in licorice representation. Water is in aqua, and chloride ions are blue spheres. The average locations of the centers of mass of phosphorus atoms shown in tan are at ± *z*_P_ = 17.35 Å.

Methionine at position 35 in Aβ25–35 (Figure 1a,b) may readily oxidize, incorporating an oxygen atom into its side chain.^42,43^ Methionine is apolar amino acid, and the resulting methionine sulfoxide (MetO) is strongly hydrophilic, whose polarity approaches those of glutamine or asparagine.^44^ Moreover, oxidized species may account for 10 to 50% of all Aβ peptides in the brain.^45,46^ Not surprisingly, oxidation radically changes the Aβ25–35 properties. For example, oxidized Aβ25–35 and oxAβ25–35 reveal no toxicity within 6 h of incubation.^41^ Additionally, this post-translational modification reduces Aβ25–35 aggregation propensity, as measured by thioflavin-T fluorescence or high-performance liquid chromatography.^47^ Previous studies of methionine oxidation in a full-length Aβ suggested that it causes the disorder in the peptide C-terminus and reduces its helical propensity.^48–52^ Furthermore, our previous simulations and application of the MM-GBSA method have revealed that oxidation reduces Aβ10–40 binding affinity to the DMPC bilayer, causes peptide expulsion from the bilayer core, and decreases the disorder in lipid structure around the bound peptide.^52^ However, it is unclear if these structural consequences of oxidation are applicable to Aβ25–35, and if so, what are the reasons for changes in binding affinity? Their study is also important because oxidation impacts Aβ25–35 aggregation propensity, as shown in our recent investigation.^53^

To address the questions above, we report in this paper the application of relative FEP/REST all-atom molecular dynamics probing the changes in the free energy of binding of Aβ25–35 to the DMPC lipid bilayer caused by Met35 oxidation. Our objective is 2-fold. First, we test the applicability of the FEP/REST methodology to the challenging binding problem presented by Aβ25–35 bound to the lipid bilayer. Not only is the peptide likely to form a multitude of binding poses and structures within the bilayer, but it is also expected to switch them upon oxidation. Second, we characterize the changes in the Aβ25–35 binding energetics and connect them with those in Aβ and lipid structures.

## Models and Methods

### All-Atom Explicit Solvent Model

Two simulation systems shown in Figure 1 were designed: one with Aβ25–35 bound to the lipid bilayer and the second featuring the peptide in lipid-free water. Specifically, the first simulation system included two Aβ25–35 peptides, 98 dimyristoylphosphatidylcholine (DMPC) lipid molecules, which form a bilayer with 49 lipids per leaflet, 4356 water molecules, and two chloride counterions. The initial dimensions of the first system were about 57 × 57 × 75 Å. The second, lipid-free system contained a single Aβ25–35 peptide, 1593 water molecules, and one chloride counterion. The initial dimensions of the second system were about 40 Å × 40 Å × 40 Å. Both systems used the dual topology method^54^ to represent simultaneously a “wild-type” (reduced) Aβ25–35 (wtAβ25–35) and its oxidized variant (oxAβ25–35) within the same simulation. Figure 1d,e illustrates a dual topology Aβ25–35, which features two reduced and oxidized side chains of methionine at position 35. The part of the peptide involved in the alchemical transformation includes the entire Met side chain. Adding oxygen to Met turns this hydrophobic residue into polar (Figure 1b). The total number of atoms, including alchemical ones, was 24,902 and 4953 in the first and second simulation systems.

The DMPC bilayer was selected because it is well characterized experimentally and was used in our previous simulations of reduced and oxidized Aβ peptides.^52,53,55^ In the first system, Aβ25–35 monomers were placed on different sides of the bilayer. This symmetric design has two advantages, as it doubles the conformational sampling and reduces the differences in pressure profiles in the leaflets.^56^ This bilayer design is not without limitations, because it may cause correlations in charge distributions across leaflets in anionic bilayers.^57^ To represent Aβ25–35, we used the all-atom CHARMM22 force field with CMAP corrections,^58^ whereas the all-atom CHARMM36 force field was used for DMPC lipids^59^ (see Supporting Information). Water was represented with the modified TIP3P model.^60,61^ The force field parameters for oxidized methionine were extracted from DMSO parametrization.^62,63^ Its force field topology and diastereomeric form were taken from the Jas and Kuczera study.^64^ Consequently, methionine sulfoxide has an R diastereomeric form. We utilize this form of oxidized Met because no clear evidence for natural preference for its enantiomeric form is known.^42,48^ Aβ25–35 N- and C-termini were acetylated and amidated, respectively.

### FEP/REST Simulations

To perform alchemical computations of the changes in binding free energy ΔΔ*G*_b_ occurring upon oxidation, we define the thermodynamic cycle in Figure 2a. Then, ΔΔ*G*_b_ is written as

1 $$\Delta \Delta G_{b}=\Delta G_{b}\left(ox\right)-\Delta G_{b}\left(wt\right)=\Delta G_{bl}\left(wt\to ox\right)-\Delta G_{w}\left(wt\to ox\right)$$

where Δ*G*_b_ is the free energy of binding, and Δ*G*_bl_ or Δ*G*_w_ are the free energy differences caused by alchemical transformation wt → ox in Aβ25–35 bound to the bilayer and immersed in lipid-free water, respectively. Since there are two peptides undergoing alchemical transformation in the bilayer system, Δ*G*_bl_(wt → ox) is equal to the free energy change in this system divided by two.

**Figure 2:**
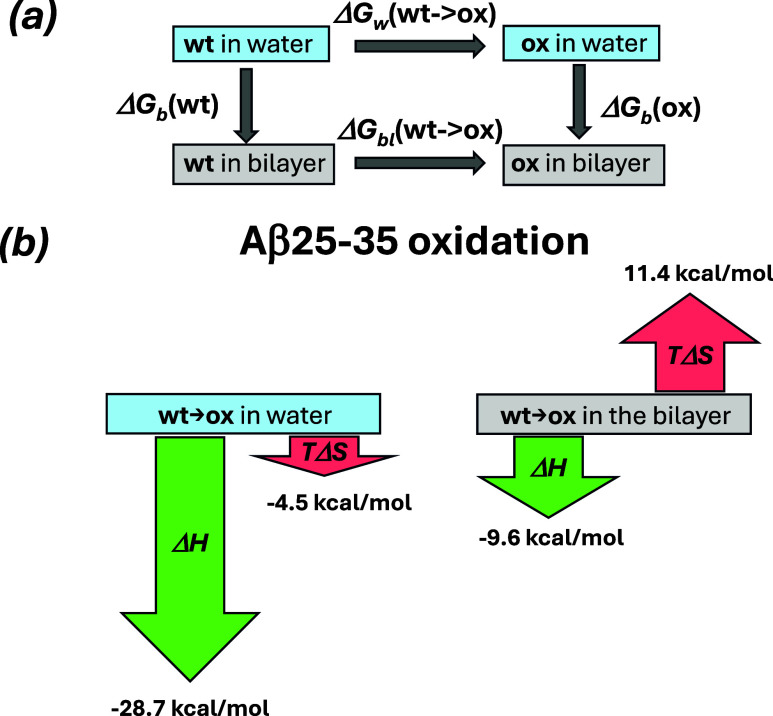
(a) Thermodynamic cycle used to compute the changes in binding free energy ΔΔ*G*_b_. (b) Diagram summarizing the changes in system’s enthalpy (in green) and entropy (in red) due to oxidation alchemical transformations in lipid-free water and in the DMPC bilayer. The lengths of arrows reflect the respective values from Table 1.

To compute the change in the free energy ΔΔ*G*_b_ of Aβ25–35 peptide binding to the DMPC bilayer caused by oxidation, we used all-atom isobaric–isothermal free energy perturbation (FEP) simulations coupled with the replica-exchange with the solute tempering (REST) algorithm. Because FEP/REST implementation is well documented in our prior publications,^24,65^ we provide below its brief overview. FEP/REST utilizes *R* replicas simulated in parallel at *R* conditions. Each condition *m* is characterized by a temperature *T*_*m*_ and a coupling factor λ_*m*_ (0 ≤ *m* ≤ *R* – 1), which controls the “expression” of wt and ox forms of Aβ25–35. In contrast to original the REST formulation,^17^ we applied Hamiltonian scaling to the interactions between solvent atoms and between solvent and solute. In all simulations, Aβ25–35 peptides and counterions were considered as the solute, whereas the rest of the system, which includes lipids (if present) and water, was treated as the solvent. Thus, the replicas are simulated at the temperature *T*_*m*_, but the Hamiltonian scaling reduces the effective temperature of the solvent to *T*_0_. Then, the system enthalpy at condition *m* is

$$H_{m}=\left(1-\lambda_{m}\right)E_{wt}+\left(1-\lambda_{m}\right)\sqrt{\frac{\beta_{0}}{\beta_{m}}}E_{wt,solv}+\lambda_{m}E_{ox}+\lambda_{m}\sqrt{\frac{\beta_{0}}{\beta_{m}}}E_{ox,solv}+E_{A\beta 0}+\sqrt{\frac{\beta_{0}}{\beta_{m}}}E_{A\beta 0,solv}+\frac{\beta_{0}}{\beta_{m}}E_{solv}+PV$$ 2

where *E*_wt_, *E*_ox_, *E*_wt,solv_, and *E*_ox,solv_ are the energies of alchemical parts of the peptide and their interactions with the solvent, respectively, *E*_Aβ0_ and *E*_Aβ0,solv_ are the energies of the non-alchemical part of Aβ25–35 and its interaction with the solvent, *E*_solv_ is the energy of the solvent, and β_*m*_ = 1/*R*_c_ *T*_*m*_ (*R*_c_ is a gas constant). If *m* = 0 and λ_0_ = 0, eq 2 solely represent wtAβ25–35, whereas at *m* = *R* – 1 and λ_*R*–1_ = 1, it represents the oxidized system. At 0 < λ < 1, partially reduced and oxidized peptides coexist without interacting. It is worth noting that to prevent disintegration of alchemical Met into ideal atoms at λ_0_ = 0 and λ_*R*–1_ = 1, the oxidized or, respectively, reduced bonded terms were preserved. According to the Metropolis criterion, replicas *r* and *r* + 1 at the conditions (*T*_*m*_, λ_*m*_) and (*T*_*m*+1_, λ_*m*+1_) are exchanged with the probability ω = min­(1, e^–Δ^), where Δ = β_*m*_(*H*_*m*_(*x*_*r*+1_) – *H*_*m*_(*x*_*r*_)) + β_*m*+1_(*H*_*m*+1_(*x*_*r*_) – *H*_*m*+1_(*x*_*r*+1_)), *x*_*r*_ and *x*_*r*+1_ are the coordinates of replicas *r* and *r* + 1. Monte Carlo simulations in the condition space implement a random walk of replicas over the conditions (*T*_*m*_, λ_*m*_). Replica exchanges were attempted every 2 ps.

FEP/REST simulations were performed using the program NAMD3 updated to permit FEP simulations with GPUs.^66^ As in our previous FEP/REST simulations, temperature was controlled using Langevin dynamics with a damping coefficient of 5 ps^–1^. Pressure was kept at 1 atm with the Langevin piston method. Van der Waals interactions were switched off in the interval from 8 to 12 Å, whereas electrostatic interactions were computed with Ewald summation. The integration step was set to 1 fs. Covalent bonds between nonwater hydrogen and heavy atoms were constrained with the ShakeH algorithm. SETTLE was applied to keep water molecules rigid. In all, we used *R* = 21 replicas for bilayer and lipid-free water simulations. For both systems, REST temperatures were geometrically distributed in the range from *T*_0_ = 330 K to *T*_(*R*–3)/2_ = 440 K for the first 10 conditions and from *T*_(*R*–1)/2_ = 429 K to *T*_*R*–1_ = 330 K for the last 11 conditions. The coupling parameter λ_*m*_ followed the temperatures *T*_*m*_ increasing the interval between midrange λ_*m*_ values. The complete list of (*T*_*m*_, λ_*m*_) conditions is given in Supporting Information Tables S1 and S2. Van der Waals interactions of reduced and oxidized Met were scaled in the range 0 ≤ λ_*m*_ ≤ 1. Electrostatic interactions of reduced and oxidized Met were canceled or ignited within the compressed schedule, i.e., within 0 ≤ λ_*m*_ ≤ 0.5 or 0.5 ≤ λ_*m*_ ≤ 1, respectively. To prevent end-point “catastrophe”, a soft-core van der Waals shifting coefficient of 5 Å^2^ was used.^67^ Thus, according to Tables S1 and S2, reduced and oxidized Aβ25–35 peptides are “fully expressed” at physiological conditions in the bilayer or lipid-free water, as prescribed for the accurate estimation of ΔΔ*G*_b_.

In all, we generated three FEP/REST trajectories for both simulation systems. Simulations of each replica in each trajectory used unique initial conditions. To prepare them, we first energy-minimized the wtAβ25–35 system and heated it to 310 K via velocity reassigning. After heating, we performed NPT FEP equilibration simulations, in which the conditions *m* were incremented with the period of 1 ps until we reached the condition of oxidized peptide. These preparatory simulations were repeated for each trajectory and used to initialize FEP/REST simulations. The bilayer simulations totalled 18.9 μs of sampling, whereas lipid-free water simulations have collected 1.26 μs. Following the convergence analysis presented in Supporting Information, we retained as equilibrated 1.512 μs for the bilayer simulations and all 1.26 μs of sampling in lipid-free water. The free energy changes along alchemical transformations in the bilayer Δ*G*_bl_ and in water Δ*G*_w_ were computed using the weighted histogram method.^68^ This method also allows us to compute enthalpic changes and the changes in van der Waals, electrostatic, and bonded energies because all of them are known for any given simulation snapshot. The entropic term was obtained by subtracting the free energy from the enthalpy.

### Computation of Structural Quantities

The interactions between amino acids and lipids were probed by computing the contacts between the centers of mass of amino acid side chains and the lipid structural groups defined in Figure 1c. A contact occurs if the distance between their centers of mass is less than 6.5 Å. To determine the secondary structure of Aβ25–35, we used the program STRIDE.^69^ A helical propensity < *H*(*i*) > is a probability for an amino acid *i* to adopt α-, 3_10_-, or π helices. Positions of Aβ25–35 amino acids in the DMPC bilayer were mapped by the probabilities *P*(*z*; *i*) for an amino acid *i* center of mass to occur at a distance *z* from the bilayer midplane. A similar probability distribution, *P*(*z*_com_), reported the position of the peptide center of mass *z*_com_. An amino acid or a peptide was considered inserted into the bilayer if its center of mass occurs below *z*_P_ = 17.35 Å, the average position of the center of mass of phosphorus atoms in a leaflet. The structure of the lipid bilayer was assessed by the volume number density of bilayer heavy atoms, *n*_l_(*r*, *z*), where *r* is the distance to the peptide center of mass and *z* is the distance to the bilayer midplane. We also defined the number density of water as *n*_w_(*r*, *z*). Using *n*_l_(*r*, *z*) and *n*_w_(*r*, *z*), the bilayer boundary *z*_b_(*r*) was computed as described.^55^ We further defined the surface number density of lipids *n*_s_ using the (*x*, *y*) positions of their P atoms. To investigate the impact of Aβ25–35 binding on the bilayer, we classified lipids as near if their P atom occurs within the distance *r* ≪ *R*_g_ > from the peptide center of mass, where < *R*_g_ > = 6.5 Å is the peptide radius of gyration. Because < *R*_g_ > for wtAβ25–35 and oxAβ25–35 differ by ≈0.1 Å, the boundary of the near region does not depend on peptide oxidation. DMPC lipids occurring within *r* > 24 Å were considered distant. The boundary of the distant region corresponds to the onset of the constant *z*_b_(*r*). Thinning of the bilayer Δ*D* was assessed by computing the thickness of the bilayer in the distant and near regions. The bilayer thickness *D* was defined as the distance between the boundaries *z*_b_ in opposite leaflets. The peptide tilt in the bilayer was described by the angle γ, which was defined between the bilayer normal and the vector connecting the first and last peptide C_α_ atoms. To compute the number of water molecules in a solvation shell < *N*_w_ >, we consider those for which oxygen atoms are no more than 3.75 Å away from any peptide heavy atom. All reported structural quantities represent the averages typically denoted as <..> computed over the equilibrium sampling at the wild-type condition (*T*_0_ = 330 *K*, λ_o_ = 0) or at the oxidized condition (*T*_*R*–1_ = 330 *K*, λ_*R*–1_ = 1). A slightly elevated temperature of 330 K accelerates sampling without altering system properties.^70^

### Conformational Ensembles and Clustering

Clustering of peptide conformations in the DMPC bilayer was performed using the method of Daura et al.^71^ Prior to peptide clustering, pairs of Aβ25–35 structures were aligned based on minimal root mean-squared deviation (RMSD). The algorithm performing the alignment of Aβ25–35 poses^72^ was modified to exclude the contribution of peptide displacement in the (*x*, *y*) plane to RMSD. The distributions of RMSDs between peptide poses were computed, and clusters were defined with the RMSD cutoff *R*_0_ = 7.0 Å for wtAβ25–35 and oxAβ25–35. The cutoff values were selected by retaining the clusters with a minimum population of 10% and increasing *R*_0_ up to the value, which provided matching between the clusters and inserted states observed in *P*(*z*_COM_) (see Supporting Information for details). A total of 7200 poses were collected for each peptide.

## Results and Discussion

### Oxidation Reduces Aβ25–35 Affinity to the DMPC Bilayer

Using FEP/REST simulations, we evaluated the changes in the free energy of binding Δ*G*_b_ of Aβ25–35 peptide to the DMPC bilayer caused by Met35 oxidation. To this end, we used the thermodynamic cycle embodied in Figure 2a and computed ΔΔ*G*_b_ using eq 1. In addition, we analyzed the free energy changes Δ*G*_bl_(wt → ox) and Δ*G*_w_(wt → ox) occurring upon alchemical transformation in the peptide placed in the bilayer and in lipid-free water. Importantly, we decomposed these changes into enthalpic and entropic components and considered individual energetic terms contributing to the enthalpic changes. The respective data are summarized in Table 1.

**Table 1:** Energetics of Oxidative Alchemical Transformations in Aβ25-35

term^a^	Δ_w_ ^b^,^e^	Δ_bl_ ^c^,^e^	ΔΔ^d^,^e^
*G*	–24.2 ± 0.1	–21.0 ± 0.1	3.2 ± 0.3
*H*	–28.7 ± 0.7	–9.6 ± 3.6	19.1 ± 3.2
*TS*	–4.5 ± 0.6	11.4 ± 3.6	15.9 ± 3.1
*E*_el_	–30.8 ± 0.6	–13.7 ± 2.7	17.1 ± 2.4
*E*_vdw_	1.7 ± 0.7	0.9 ± 1.0	–0.8 ± 1.5
*E*_conf_	0.4 ± 0.2	3.1 ± 0.7	2.7 ± 0.8

a The terms in the table are computed per peptide and in kcal/mol.

b Changes in respective terms caused by wt → ox alchemical transformation in lipid-free water.

c Changes in respective terms caused by wt → ox alchemical transformation in the DMPC bilayer.

d Changes in binding energetics caused by wt → ox alchemical transformation.

e In (b–d) wtAβ25–35 is taken as the reference.

We first analyze thermodynamic changes occurring in the peptide bound to the DMPC bilayer. According to Table 1, Δ*G*_bl_ = −21.0 kcal/mol unequivocally indicates that oxidation is energetically highly favorable, resulting in a considerable decrease in free energy. Its decrease is driven by both enthalpic and entropic factors signified in the enthalpic decrease Δ*H*_bl_ (−9.6 kcal/mol) and entropic gain *T*Δ*S*_bl_ (11.4 kcal/mol). These energetic changes are illustrated in Figure 2b. Thus, due to the alchemical transformation, wt → ox Aβ25–35 forms more favorable interactions, and the system acquires additional conformational freedom. Interestingly, Δ*H*_bl_ and *T*Δ*S*_bl_ provide almost equal contributions to Δ*G*_bl_. suggesting that both factors are equally important. Enthalpic gains upon oxidation have three highly uneven contributions coming from the changes in electrostatic, van der Waals, and conformational energies. Gains in electrostatic energy Δ*E*_el_ are highly favorable and clearly dominant. There is some straining of bonded terms and a negligible loss in *E*_vdw_. Thereby, enthalpic gains are driven by electrostatic interactions.

Next, we examine thermodynamic changes occurring upon alchemical transformation in lipid-free water. According to Table 1, this transformation leads to an even more considerable decrease in the free energy (Δ*G*_w_ = −24.2 kcal/mol). Table 1 and Figure 2b show that this outcome results from the combination of two opposing factors: a strong enthalpic gain (Δ*H*_w_ = −28.7 kcal/mol) coupled with relatively minor entropic losses (*T*Δ*S*_w_ = −4.5 kcal/mol). Thus, oxidation in water creates favorable interactions between Aβ and water but also restrains the system’s structural freedom. Since enthalpic gains strongly dominate entropic losses by more than 6-fold, the former is a driver of the energetic changes. Similar to the alchemical transformation in the bilayer, enthalpic gains in lipid-free water have one primary source, namely, electrostatic interactions. Their change Δ*E*_el_ = −30.8 kcal/mol is highly favorable, dominating other energetic terms. Hence, in both environments oxidation is thermodynamically highly favorable, but enthalpic gains, primarily coming from electrostatic interactions, are about 3-fold larger in water than in the bilayer. The two environments lead to differing entropic changes. Whereas oxidation results in strong entropic gains in the bilayer, the opposite is seen in lipid-free water.

Compiling the above analysis together and using the thermodynamic cycle in Figure 2a, energetic gains/losses in Figure 2b, and eq 1, we deduce the changes in binding energetics caused by methionine oxidation. The respective data are listed in Table 1. The overall change in binding free energy is ΔΔ*G*_b_ = 3.2 kcal/mol >0, which suggests that oxidation reduces the binding affinity of Aβ25–35 for the DMPC bilayer. This is the central result of our study. Interestingly, a decrease in binding affinity results from a partial cancellation of two large but opposing factors. Oxidation makes binding dramatically less enthalpically favorable (ΔΔ*H*_b_ = 19.1 kcal/mol >0). At the same time, oxidation mitigates entropic losses upon binding (*T*ΔΔ*S*_b_ = 15.9 kcal/mol >0). In the end, the entropic gain falls short of compensating for the enthalpic losses upon binding. The structural factors rationalizing the energetic changes are examined below.

### Oxidation Changes the Conformational Ensemble of Aβ25–35 in the DMPC Bilayer

To rationalize the changes in Aβ25–35 binding energetics, we examined the effect of oxidation on the peptide structures, its interactions with the environment, and bilayer properties. wtAβ25–35 in the DMPC bilayer: Within the DMPC bilayer, wtAβ25–35 adopt a helical structure with the average helical propensity of < *H* > = 0.62 ± 0.02. Figure 3a shows that the residue-specific helical propensity < *H*(*i*) > peaks in the C-terminus for *i* = 30 to 34. The radius of gyration of wtAβ25–35 is < *R*_g_ > = 6.5 ± 0.3 Å. To map the position of the wtAβ25–35 peptide within the lipid bilayer, we first computed the probability distribution *P*(*z*_com_) of the peptide center of mass *z*_com_. Figure 4a demonstrates that *P*(*z*_com_) for the reduced peptide is bimodal, implicating two bound states. Using the minimum of *P*(*z*_com_) at *z*_com_ = 7.5 Å we distinguish the inserted state (I) with *z*_com_ > 7.5 Å and the deeply inserted state (DI) with *z*_com_ < 7.5 Å. The probabilities for wtAβ25–35 to occur in I and DI are 0.61 ± 0.16 and 0.39 ± 0.16, respectively. On average, the peptides within I are located at the distance *z*_com_ = 13.0 ± 0.1 Å from the bilayer midplane, confirming that they are inserted in the bilayer (see Models and Methods). The DI peptides are positioned much deeper in the bilayer, with *z*_com_ = 2.8 ± 0.2 Å. To verify the partitioning of wtAβ25–35 peptides between I and DI, we performed their structural alignment and clustering following the procedure described in the Models and Methods. The results provided in Supporting Information show that with *R*_c_ = 7.0 Å, the peptides can be grouped into two clusters, which closely reproduce I and DI states. The centroids of these clusters are displayed in Figure 4a. Interestingly, the I and DI states have different helical propensities. For reduced peptides in I, *H* = 0.50, but the helix fraction increases to 0.82 in the DI state. Thus, most wtAβ25–35 populate the I state with moderate helical propensity, but about a third of peptides are deeply inserted and acquire additional helical structure.

**Figure 3:**
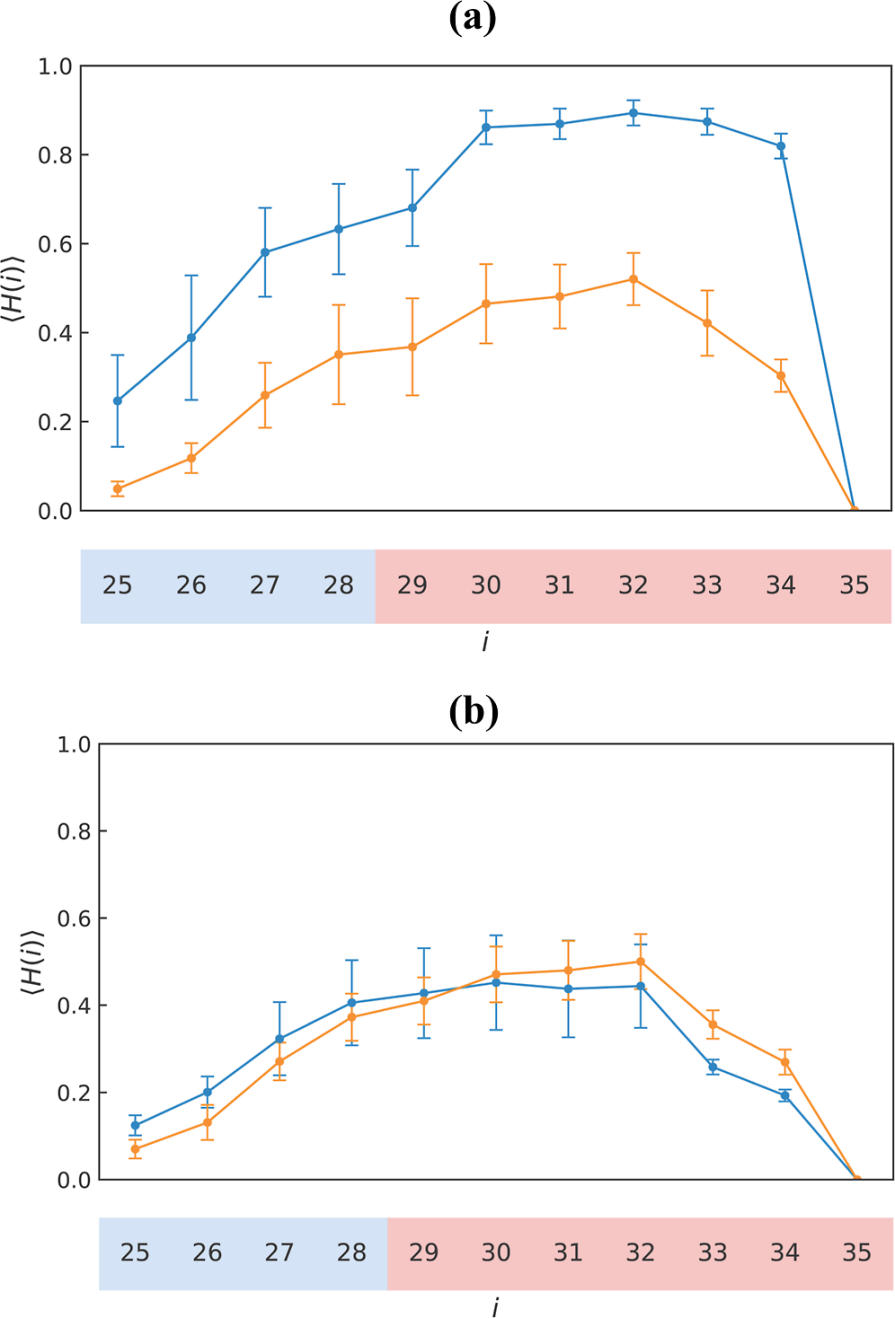
Fraction of helical structure < *H*(*i*) > sampled by Aβ25–35 residues *i* in the DMPC bilayer (a) and in lipid-free water (b). In both panels, blue and orange lines represent reduced and oxidized Aβ25–35. Standard errors are shown by vertical bars. The figure indicates that oxidation drastically reduces helical propensity in the peptide bound to the DMPC bilayer but leaves it largely intact in lipid-free water.

**Figure 4:**
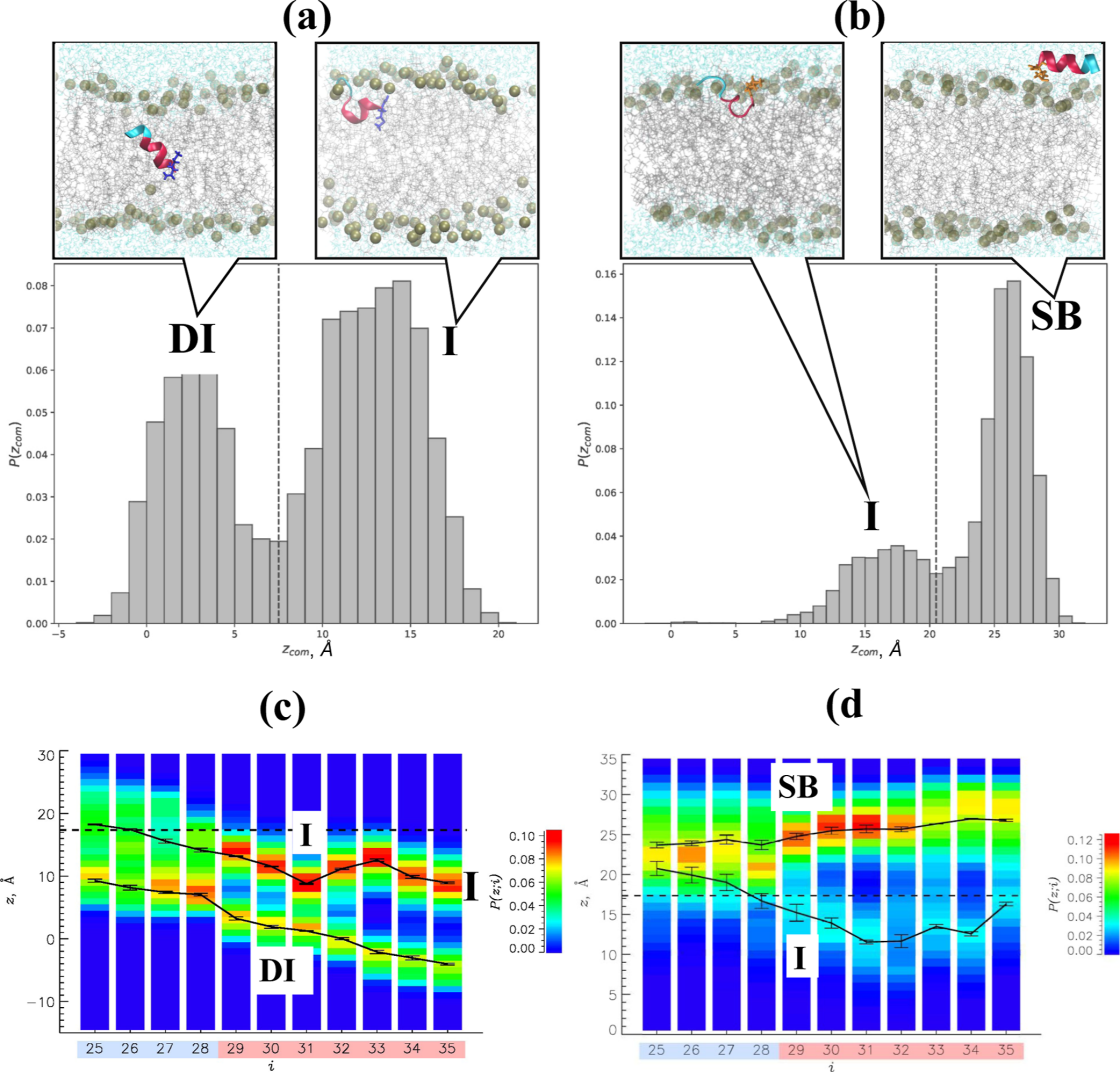
Probability distributions *P*(*z*_com_) of the center of mass positions *z*_com_ of reduced (a) and oxidized (b) Aβ25–35 peptides. The value of *z*_com_ = 7.5 Å, marked by a dashed line, separates the inserted (I) and deeply inserted (DI) wtAβ25–35 states. The surface-bound (SB) and inserted (I) states for oxAβ25–35 are partitioned at *z*_com_ = 20.5 Å, marked by a dashed line. The centroids of conformational clusters representing the bound states are shown. Structural representation follows that of Figure 1d, apart from the second peptide being omitted for clarity. The probabilities *P*(*z*; *i*) for the centers of mass of amino acids *i* to occur at the distance *z* from the bilayer midplane are presented for wtAβ25–35 (c) and oxAβ25–35 (d). The average positions of amino acids *z*(*i*) in the bound peptide states are shown by thick black lines with errors. The average position of the center of mass of phosphorus atoms in a leaflet is given by the dashed line. The figure demonstrates that the populations of reduced and oxidized peptides fall into two distinct states with different depths of bilayer penetration and orientations.

To probe the allocation of individual amino acids in the bilayer, we considered the probabilities *P*(*z*; *i*) for the centers of mass of amino acids *i* to occur at the distance *z* from the bilayer midplane (Figure 4c). These probabilities confirm the existence of two inserted states but offer additional insights. The average positions of amino acid centers of mass *z*(*i*) in the I state reveal a relatively flat orientation of wtAβ25–35 in the bilayer. Indeed, the tilt angle of I peptides γ is 128°. In contrast, the peptides assigned to the DI state are tilted at γ = 153°. Strikingly, because of a deep peptide insertion in the bilayer and tilted orientation, its C-terminus crosses the midplane and emerges in the opposite leaflet. It is worth noting that this deep penetration of Aβ25–35 in the bilayer may trigger peptide dimerization with a probability of 0.15. However, extensive checks presented in Supporting Information suggest that these transmembrane aggregates do not appreciably change the energetic and conformational properties of Aβ25–35.

The binding interactions formed by wtAβ25–35 with the DMPC bilayer were probed by computing the contacts between amino acids and lipid groups. The numbers of binding contacts < *C*_b_(*i*) > formed by amino acids *i* are shown in Figure 5a. Overall, the peptide forms < *C*_b_ > = 17.2 ± 0.8 binding contacts, and the amino acids contributing the most to binding (the top quarter) are Lys28 (<*C*_b_(28) > = 2.2 ± 0.2), followed by Asn27 (1.9 ± 0.2), Ser26 (1.9 ± 0.2), and Leu34 (1.7 ± 0.2). Thus, binding interactions are mostly localized at three N-terminal polar amino acids. In fact, the strongest contact is an electrostatic interaction between cationic Lys28 and negatively charged phosphate group L2 (Figure S6 in Supporting Information). Although wtAβ25–35 is inserted in the bilayer, it is still partially hydrated. The peptide solvation shell contains < *N*_w_ > = 34 ± 0 water molecules, of which 4 ± 0 coordinate Met35.

**Figure 5:**
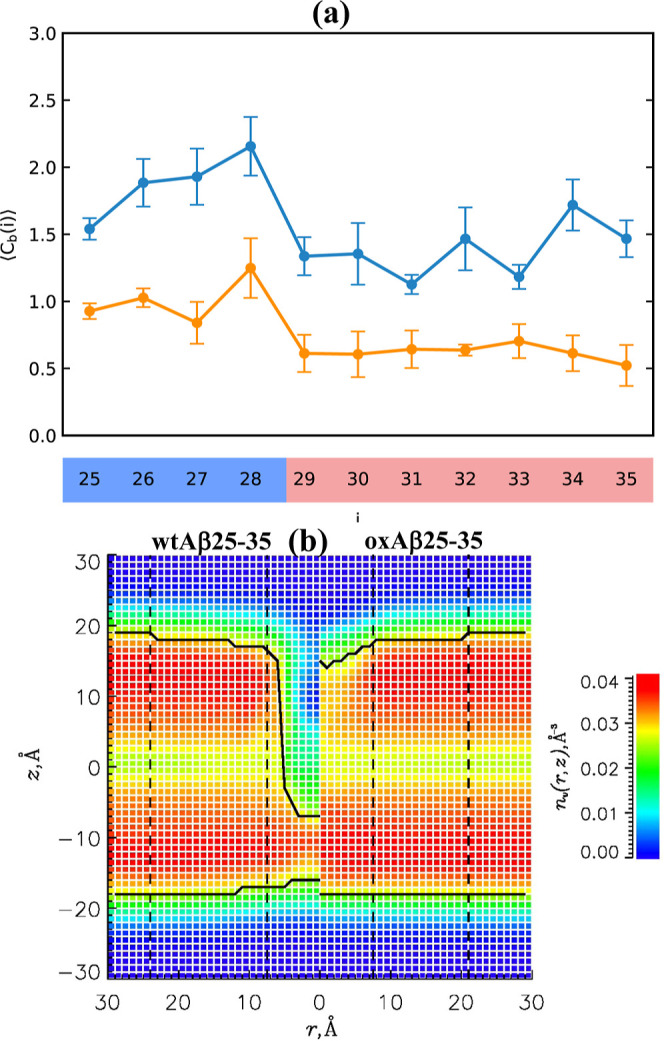
(a) Number of binding contacts < *C*_b_(*i*) > formed by amino acids *i* with lipid groups. The data in blue and orange represent reduced and oxidized Aβ25–35 peptides. (b) Volume number densities of heavy lipid atoms *n*_l_(*r*, *z*) as a function of the distance *r* to the peptide center of mass and the distance *z* to the bilayer midplane. Left and right sections of the plot present *n*_l_(*r*, *z*) for wtAβ25–35 and oxAβ25–35, respectively. Continuous thick black lines mark the bilayer boundaries *z*_b_(*r*). The panel shows that Met35 oxidation drastically alleviates bilayer disruption.

One may expect that a deep insertion of the reduced peptide into the DMPC bilayer disorders the lipids. Figure 5b shows the number density of heavy lipid atoms, *n*_l_(*r*, *z*), where *r* is the distance to the peptide center of mass, and *z* is the distance to the bilayer midplane. The density map shows a deep void occurring within the Aβ25–35 binding footprint. In fact, binding of the peptide causes bilayer thinning by Δ*D* = 19.0 ± 3.5 Å. Bilayer thinning is accompanied by a steep, one-third reduction in the volume number density of heavy lipid atoms *n*_v_ between the distant (*n*_v_ = 0.034 ± 0.000 Å^–3^) and near (0.022 ± 0.000 Å^–3^) regions. Simultaneously, a more than 30% depletion of the lipid surface number density *n*_s_ is observed, from 0.015 ± 0.000 Å^–2^ in the distant region to 0.010 ± 0.001 Å^–2^ in the near region.

#### oxAβ25–35 in the DMPC Bilayer

The helical fraction < *H* > for the oxidized peptide is 0.30 ± 0.02. Figure 3a shows that wt → ox transformation uniformly depresses the helical propensity < *H*(*i*) > for all amino acids *i*. However, despite helix destabilization, the peptide radius of gyration remains virtually unchanged (<*R*_g_ > = 6.6 ± 0.4 Å). The probability distribution *P*(*z*_com_) of the oxidized peptide center of mass *z*_com_ in Figure 4b is bimodal, implicating two bound states. Using *z*_com_ = 20.5 Å to separate them, we identify the surface bound (SB) state with *z*_com_ > 20.5 Å and the inserted state (I) with *z*_com_ < 20.5 Å. The oxidized peptide resides in the SB state with the probability 0.73 ± 0.10, while it samples the I state less frequently with the probability 0.27 ± 0.10. In the SB state, oxAβ25–35 is located, on average, at *z*_com_ = 25.8 ± 0.3 Å that is well above the average position of phosphorus atoms at *z*_P_ = 17.35 Å, thus justifying the state name. Within I, the peptide center of mass occurs at *z*_com_ = 16.1 ± 0.4 Å from the bilayer midplane, implicating a shallow insertion of oxAβ25–35 in the bilayer. As for wtAβ25–35, the oxidized peptides were structurally aligned and clustered. We show in Supporting Information that the cutoff *R*_0_ = 7.0 Å allows us to perfectly map the two peptide clusters into SB and I states (Figure 4b). The helical propensities of SB and I states are both low (0.34 and 0.19). The probabilities *P*(*z*; *i*) in Figure 4d probing the positions of individual amino acids in the bilayer show that in the SB state, they occur well above the phosphorus atoms. A trace of probabilities just below the phosphorus positions constitutes the I state. Both states exhibit a fairly flat peptide profile, suggesting a nearly horizontal orientation of oxAβ25–35. This conclusion is supported by the computations of tilt angles γ, which are 76° for SB and 112° for I.

The binding interactions between oxAβ25–35 and the DMPC bilayer are presented in Figure 5a. The total number of binding contacts is < *C*_b_ > = 8.4 ± 1.3, and the amino acids which contribute the most to binding (the top quarter) are Lys28 (<*C*_b_(28) > = 1.3 ± 0.2) and Ser26 (1.0 ± 0.1). Thus, oxAβ25–35 binding interactions are dominated by the polar contacts formed by the N-terminus (see also Figure S6). The numbers of water molecules < *N*_w_ > forming the solvation shells around oxAβ25–35 and Met35 are 63 ± 0 and 14 ± 0, respectively. The disruption of the bilayer structure by oxAβ25–35 is evaluated in Figure 5b. The density map *n*_l_(*r*, *z*) shows a shallow indentation caused by oxAβ25–35 binding, which results in minor bilayer thinning Δ*D* = 3.4 ± 1.0 Å. Bilayer thinning is accompanied by a small decrease in the lipid surface number density from *n*_s_ = 0.015 ± 0.000 Å^–2^ in the distant region to 0.012 ± 0.001 Å^–2^ in the near. However, there is virtually no change in the volume number density, *n*_v_, which maintains its distant value of 0.034 ± 0.000 Å^–3^.

By comparing the ensembles of reduced and oxidized Aβ25–35 peptides bound to the DMPC bilayer, we draw the following conclusions. First, Met35 oxidation causes a dramatic, more than 2-fold, drop in the peptide helical fraction < *H* > and uniformly depresses the helical propensity < *H*(*i*) > for all amino acids. Second, wt → ox transformation expels the peptide from the bilayer core to its surface. We observed that wtAβ25–35 samples two states, inserted I and deeply inserted DI, and the average position of its center of mass is < *z*_com_ > = 9.0 ± 1.7 Å (averaged over both states). Conversely, oxAβ25–35 populates predominantly the surface bound SB state as well as the less frequently inserted I state. The average position of its center of mass is < *z*_com_ > = 23.2 ± 1.2 Å, which is more than 14 Å above wtAβ25–35, placing the oxidized peptide in the bilayer–water interface. Third, Met35 oxidation compromises the binding interactions, reducing them approximately in half. However, for both peptide forms, the anchor of binding interactions is the polar N-terminus. Fourth, Aβ25–35 oxidation dramatically facilitates peptide hydration. The number of water molecules forming the peptide solvation shell doubles, whereas the number of those hydrating Met35 increases almost 4-fold. Fifth, consistent with the peptide expulsion and weaker binding interactions, wt → ox transformation sharply alleviates the disruption in the bilayer structure. This change is manifested in an almost 6-fold smaller bilayer thinning caused by oxAβ25–35 and a minor change in lipid number density.

#### Aβ25–35 Peptide in Lipid-Free Water

Strikingly, oxidation produces a markedly weak impact on the Aβ25–35 helical structure in water. In fact, the overall helical fraction < *H* > in wtAβ25–35 is 0.27 ± 0.05 compared to 0.28 ± 0.04 for oxAβ25–35. The corresponding variations in < *R*_g_ > are also within the sampling error (6.7 ± 0.2 vs 6.9 ± 0.1 Å). Alchemical transformation moderately facilitates peptide-water interactions. The solvent accessible surface area (SASA) of Met35 increases 3%, from 320 ± 0 Å^2^ for the reduced amino acid to 329 ± 0 Å^2^ when oxidized. There is a minor increase in peptide SASA, from 1344 ± 20 Å^2^ to 1363 ± 13 Å^2^. We also computed the number of water molecules < *N*_w_ > coordinating the peptide and Met35. Upon oxidation, the peptide hydration shell adds about five extra water molecules as < *N*_w_ > increases from 64 ± 0 to 69 ± 0. Simultaneously, two extra water molecules surround Met35, for which < *N*_w_(35) > changes from 11 ± 0 to 13 ± 0. It follows from this analysis that the overall wt → ox transformation results in small structural changes in Aβ25–35. This outcome is due to the small peptide size, which prevents Aβ25–35 from folding or unfolding depending on the Met35 state.

#### Connecting Energetic and Structural Changes Caused by Oxidation

It is important to link the energetic and structural changes caused by wt → ox alchemical transformation. A sharp decrease in helical structure in the Aβ25–35 peptide bound to the DMPC bilayer due to oxidation is caused by diminished hydrophobic moment μ. In our previous publication we estimated that Met35 oxidation decreases μ from 4.4 to 3.2 kcal/mol.^53^ Furthermore, the emergence of a highly polar amino acid at position 35 destabilizes the I and DI states, in both of which the peptide is inserted into the hydrophobic bilayer core. As a result, oxidation causes expulsion of oxAβ25–35 to the bilayer surface, boosting peptide hydration. Melting of the helix around Met35 in the expelled peptide allows it to adopt a structure maximizing interactions with water. Predictably, this conformational restructuring leads to a much weaker distortion of the DMPC lipids in the bilayer caused by Aβ25–35 binding. Together, these transitions rationalize the energetic changes in the peptide + bilayer system. Strong enthalpic gain is caused by the relocation of polar oxidized Met35 from the bilayer core to the water interface, resulting in its dramatically better solvation. An increase in the system entropy is likely due to three factors - helix destabilization in oxAβ25–35, its expulsion from the bilayer, and minimal distortion of the bilayer structure by the oxidized peptide, which all taken together promote conformational freedom. The connection between energetic and structural changes in lipid-free water can also be deduced. The dramatic enthalpic gain is caused by the formation of favorable interactions between Met35 and water. Interestingly, because oxidation does not restructure the peptide in lipid-free water, this enthalpic gain is accompanied by only a moderate boost in hydration. A small but discernible decrease in entropy caused by wt → ox is likely due to capturing and restraining additional water molecules in the solvation shell of the oxidized peptide.

The above analysis may explain the changes in the Aβ25–35 binding affinity. Oxidation makes binding enthalpically less favorable because the gains in enthalpy in water far surpass those within the bilayer. Somewhat unexpectedly, this outcome is not driven by better hydration of oxidized Aβ25–35 in water compared to that in the lipid bilayer. In fact, the numbers of water molecules < *N*_w_ > in the solvation shells of oxAβ25–35 in water and bound to the bilayer differ merely by 10% (69 ± 0 vs 63 ± 0). Note also that wt → ox alchemical transformation increases the number of water molecules in the Aβ25–35 solvation shell about 2-fold in the bilayer but by less than 10% in lipid-free water. Nevertheless, Table 1 shows that the gain in electrostatic energy, a primary driver of enthalpy, is more than twice as high in water as in the bilayer. Then, although wt → ox strongly facilitates peptide hydration in the bilayer, it must also compromise electrostatic interactions elsewhere, presumably by exposing apolar amino acids to water in the SB state. We surmise that the real factor making binding less enthalpically favorable is that wt → ox transformation introduces minimal energetic frustration in water compared to the bilayer. However, oxidation also mitigates binding entropic losses, as it results in strong gains in conformational freedom within the bilayer system. In the end, entropic gain is insufficient to compensate for the enthalpic binding loss.

### Comparison with Previous Studies

In our previous studies, we evaluated the impact of Met35 oxidation on the binding of longer peptide Aβ10–40 to the DMPC bilayer.^52^ Consistent with the alchemical transformation wt → ox for Aβ25–35, oxidized Aβ10–40 exhibits lower helical propensity by about 2-fold. Oxidized Aβ10–40 predominantly binds to the DMPC bilayer via its C-terminus. Furthermore, oxidized Aβ10–40 produced 3-fold smaller bilayer thinning than the reduced peptide and caused a smaller drop in surface and volume number densities of lipids. Finally, previous REST simulations utilized the MM-GBSA method to compute the changes in Aβ10–40 binding affinity. The analysis showed that oxidation reduced the binding free energy by ΔΔ*G*_b_ = 17 ± 9 kcal/mol, i.e., oxidized Aβ10–40 has weaker binding affinity than the reduced peptide. These results argue that the impacts of oxidation on the short Aβ25–35 fragment and its longer counterpart Aβ10–40 are qualitatively similar. First, oxidation compromises the helical structure in both peptides, reduces bilayer disorder caused by binding, and reduces the binding affinity to the bilayer. Along with these similarities, there are subtle differences. Met35 oxidation redistributes binding interactions along the Aβ10–40 sequence making the C-terminus the binding anchor. This change in the binding interactions is not seen for Aβ25–35.

It is interesting to compare the impact of Met35 oxidation on Aβ25–35 monomers and dimers.^53^ We have previously shown that oxAβ25–35 dimers have lower helical propensity and are expelled from the DMPC bilayer when compared to the reduced peptides. Indeed, due to oxidation, the average helical fraction is reduced from < *H* > = 0.36 ± 0.03 to 0.32 ± 0.03. Moreover, the peptides are displaced closer to the bilayer surface by Δ*z* = 5.9 Å, which is more than twice smaller than for Aβ25–35 monomers in our work. Also, within the dimer, oxAβ25–35 peptide forms 30% fewer binding contacts with the DMPC bilayer than wtAβ25–35. Thus, while the impact of oxidation on Aβ25–35 monomers and dimers may diverge in specifics, the trends remain common: helix destabilization, peptide expulsion from the bilayer, and weaker binding affinity to the bilayer. Combining the findings for Aβ10–40, Aβ25–35 monomers and dimers, we surmise that these effects are likely to be general for Aβ species.

Finally, we compare FEP/REST sampling of wtAβ25–35 with our previous simulations of this peptide using REST.^55^ That study has reported that the peptide binding to the DMPC bilayer samples two states, surface bound, which occurred with the probability of about 0.7, and inserted, observed with the probability of ≈0.3. In the previous simulations, the N-terminus served as an anchor of binding interactions, and the peptide helical fraction was < *H* > = 0.3. Thus, FEP/REST reveals a deeper insertion of wtAβ25–35 into the DMPC bilayer with a stronger helical propensity. Both simulations, however, agree that the peptide samples the inserted state I, which in the previous simulations caused almost the same bilayer thinning (Δ*D* = 15.1 ± 0.4 Å), as seen in the current study. The reason for the discrepancy between FEP/REST and REST is 2-fold. First, FEP/REST used the final structures of REST simulations and effectively extended sampling by almost 19 μs. Second, alchemical transformation in FEP/REST features intermediate conditions around λ = 0.5 (Figure 1d), where all electrostatic interactions formed by Met35 are annihilated, making it completely apolar. These conditions promote deeper insertion of Aβ25–35 into the bilayer, enriching the states for the reduced peptide.

Reduced wtAβ25–35 has been the subject of numerous experimental studies permitting us to make comparisons with in silico results. Two NMR structures for this peptide were reported, one interacting with LiDS micelles^73^ and the other bound to SDS micelles.^74^ In those structures, a helix is observed in the sequence region between Lys28 and Leu34 with the N-terminus being disordered. Those findings are in good agreement with our data on wtAβ25–35, which reveal a stable helical state (<*H*(*i*) ≫ 0.5) for amino acids *i* = Asn27 to Leu34. Multiple experimental investigations have found that wtAβ25–35 binds to zwitterionic bilayers or vesicles formed by POPC^75^ or DLPC^76^ lipids or to weakly anionic POPC/POPS and POPC/POPG bilayers.^77–79^ Specific binding information comes from the studies of Dante et al.,^77^ which identified two peptide states upon binding to the POPC bilayer. Using the position of deuterated Leu34, the authors identified the inserted state, in which this amino acid is positioned *z* ∼ 14 Å away from the bilayer midplane. In the second state, d-Leu 34 is bilayer SB being at *z* ∼ 27 Å. Importantly, the inserted state is overwhelmingly populated, occurring with the probability of 0.86. This state approximately agrees with our dominant inserted state I, for which < *z*(34) > = 9.7 ± 2.9 Å. Because the POPC bilayer is thicker than DMPC,^80^ it is expected that Leu34 is positioned farther away from the POPC midplane than from the DMPC one. Several wtAβ25–35 states embedded in a weakly anionic 97:3 DMPC/DMPS bilayer have also been reported.^81^ About 40% of the peptide electrons were found at *z* ≲ 9 Å. This allocation of wtAβ25–35 electrons roughly corresponds to our DI state, which occurs at *z*_COM_ < 7.5 Å with the probability of 0.39. However, this agreement between experimental and computational wtAβ25–35 bound states should be viewed with caution due to difficulties in associating the peptide center of mass position with electron density.

We are not aware of biophysical studies probing the binding of oxAβ25–35 to lipid bilayers and comparing it with the wild-type form. Generally, experiments have established that methionine oxidation impairs aggregation and reduces cytotoxic propensity of full-length Aβ peptides.^49,82,83^ Since Aβ25–35 is a model of the full-length peptide, it is likely that these observations are relevant to this short fragment. Indeed, oxidized Aβ25–35 shows no cytotoxicity within 6 h of incubation.^41^ Met35 oxidation also reduces peptide aggregation propensity.^47^ The same conclusion follows from our recent REST simulations exploring the impact of oxidation on Aβ25–35 dimerization.^53^ Then, if Aβ25–35 cytotoxicity is driven by its binding to lipid membranes and subsequent aggregation, our FEP/REST simulations provide a plausible explanation for these observations based on the reduced binding affinity of oxidized Aβ25–35 to the bilayer. Additionally, our investigation offers a molecular understanding of this effect.

## Conclusions

In this study, we applied relative FEP/REST all-atom molecular dynamics to determine the changes in the free energy of binding of Aβ25–35 to the DMPC lipid bilayer caused by Met35 oxidation. From the methodological perspective, we showed that the restraint-free FEP/REST protocol delivers converged sampling of the alchemical transformation in the DMPC bilayer. In our opinion, this is an important advance because Aβ25–35 binding presents three challenges to FEP: (i) the peptide adopts multiple conformations and positions within the bilayer, (ii) alchemical transformation forces the peptide to change conformations and positions, and (iii) lipid bilayer adjusts in response to alchemical transformation. From a scientific perspective, we found that Met35 oxidation moderately reduces the peptide binding free energy by ΔΔ*G*_b_ = 3.2 kcal/mol. The decrease in ΔΔ*G*_b_ comes from a partial cancellation of two large opposing factors. Oxidation makes binding less enthalpically favorable, but it also mitigates entropic losses. The entropic gain is not strong enough to compensate for the enthalpic binding loss. The analysis identified two sources of these changes, namely, (i) a minimal energetic frustration introduced by Met35 oxidation in water compared to the bilayer and (ii) entropic gains within the bilayer system. The latter source is due to disruption of the helical structure in Aβ25–35, expulsion of the peptide from the bilayer core, and alleviating lipid disorder. Taken together, our work sheds light on the molecular mechanism by which oxidation changes Aβ25–35 properties, potentially rationalizing experimental observations on cytotoxicity.

## Supplementary Material



## Data Availability

NAMD is available at https://www.ks.uiuc.edu/Research/namd/. VMD is available at https://www.ks.uiuc.edu/Research/vmd/. Initial structures, topology files, NAMD configuration files, and analysis scripts are available at https://github.com/KlimovLab/oxAb.

## Notes

Additional details concerning the model, methods, and results (PDF)
