# Supplementary material for: Free Energy Perturbation Simulations Measure the Change in Binding Affinity of the Aβ25–35 Peptide to the Zwitterionic Bilayer Caused by Oxidation
Source: J Chem Inf Model. 2025 Oct 22;65(21):12014–26. doi: 10.1021/acs.jcim.5c02148 (PMC12606640; doi:10.1021/acs.jcim.5c02148)
Supplement: Supplementary file 1 [file ci5c02148_si_001.pdf]

Supporting Information

Free energy perturbation simulations measure the change in binding affinity of Aβ25-35 peptide to zwitterionic bilayer caused by oxidation

Xingyu Luo, Elias Khayat, Steven R. Bowers, Bryan M. Delfing, Christopher Lockhart, and Dmitri K. Klimov
School of Systems Biology, George Mason University, Manassas, VA 20110
E-mail: dklimov@gmu.edu

Selection of peptide force field: Following our previous studies we selected the all-atom CHARMM22 force field with CMAP corrections to represent Aβ25-35. This force field has demonstrated an approximate agreement of *in silico* distribution of peptide helical structure and experimental data [1-3].

FEP/REST setup: To evaluate the changes in free energy due to Aβ25-35 oxidation, we used the relative free energy perturbation method and replica exchange with solute tempering molecular dynamics (FEP/REST). The thermodynamic cycle in Fig. 2a requires two FEP/REST simulations, one for the peptide bound to the DMPC bilayer and the other in lipid-free water. Description of these FEP/REST simulations is given in Models and Methods. The bilayer simulations used R=21 conditions (T_m,λ_m), where T_m is the temperature, λ_m is the alchemical coupling factor, and m is the condition index. The list of FEP/REST conditions applied to the bilayer simulations is given in Table S1.

Table S1: List of FEP/REST conditions used in the simulations of Aβ25-35 in the DMPC bilayer.

m	T_m	λ_m
**0**	330.00	0.000
**1**	341.00	0.032
**2**	352.00	0.069
**3**	363.00	0.110
**4**	375.00	0.157
**5**	387.00	0.210
**6**	400.00	0.269
**7**	413.00	0.337
**8**	426.00	0.414
**9**	440.00	0.500
**10**	429.00	0.572
**11**	418.00	0.636
**12**	407.00	0.695
**13**	396.00	0.748
**14**	386.00	0.795
**15**	376.00	0.839
**16**	366.00	0.878
**17**	357.00	0.913
**18**	348.00	0.945
**19**	339.00	0.974
**20**	330.00	1.000

The FEP/REST simulations in lipid-free water utilized R=21 conditions (T_m,λ_m). Their list is given in Table S2.

Table S2: List of FEP/REST conditions used in the simulations of Aβ25-35 in lipid-free water.

m	T_m	λ_m
**0**	330.00	0.000
**1**	341.00	0.032
**2**	352.00	0.069
**3**	363.00	0.110
**4**	375.00	0.157
**5**	387.00	0.210
**6**	400.00	0.269
**7**	413.00	0.337
**8**	426.00	0.414
**9**	440.00	0.500
**10**	429.00	0.572
**11**	418.00	0.636
**12**	407.00	0.695
**13**	396.00	0.748
**14**	386.00	0.795
**15**	376.00	0.839
**16**	366.00	0.878
**17**	357.00	0.913
**18**	348.00	0.945
**19**	339.00	0.974
**20**	330.00	1.000

FEP/REST performance: FEP/REST technical performance was assessed following the approach adopted in our previous studies [4]. FEP/REST formalism prescribes random walks of replicas across the conditions. Fig. S1 demonstrates that FEP/REST simulations of Aβ25-35 peptide in the bilayer and lipid-free water produce largely random walk of replicas across the conditions. The figure does not reveal persistent trapping of replicas at any condition.

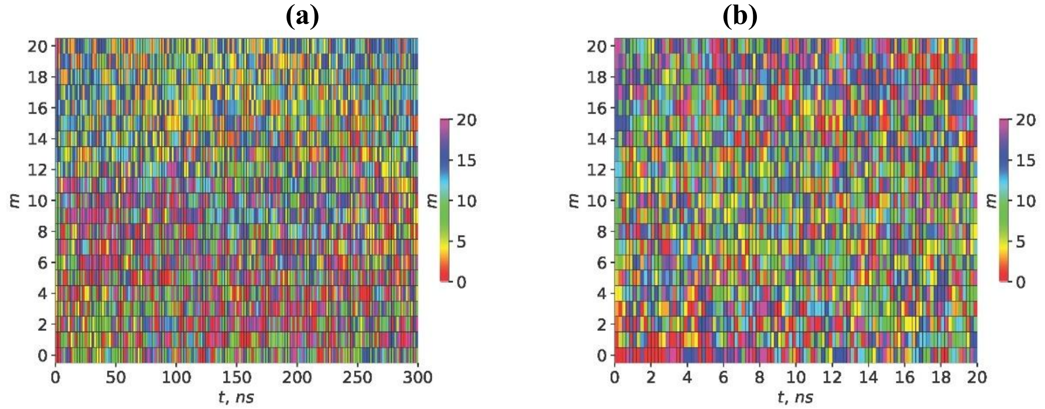

Figure S1 Traveling of replicas across conditions m in the representative trajectories from FEP/REST simulations in the DMPC bilayer (a) and lipid-free water (b). Colors are assigned to replicas at the start of the trajectory.

To probe the distribution of replicas across the conditions quantitatively, we computed the replica mixing parameter [5],

$$h(m) = 1 - \frac{\sqrt{\sum_{r=0}^{R-1} t_r^2}}{\sum_{r=0}^{R-1} t_r}, \tag{S1}$$

where m is the FEP/REST condition and t_r is the time spent by replica r at m. If FEP/REST produces a truly random distribution of replicas over R conditions, $h(m)$ assumes the maximum theoretical value $h_o = 1 - 1/R^{1/2}$. For the FEP/REST simulations in the DMPC bilayer and in lipid-free water h_o is 0.78. Fig. S2 shows that for the two FEP/REST simulations $h(m)$ approaches h_o. In fact, the average h across all m conditions is 0.71 for the FEP/REST simulations in the bilayer and 0.77 in lipid-free water. Next, Fig. S3 presents the replica exchange rates $\alpha(m)$. The average $\alpha(m)$ are 0.30 and 0.46 for the FEP/REST simulations in the bilayer and lipid-free water, respectively. These rates are approximately optimal [6]. Taken together Figs. S1-S3 suggest a good mixing of replicas across the conditions as expected from FEP/REST.

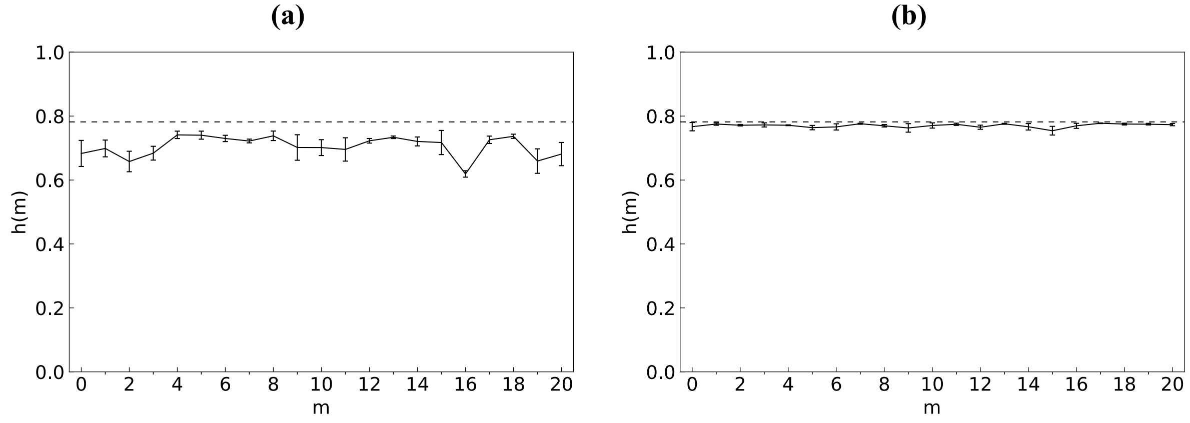

Figure S2 The replica mixing parameter $h(m)$ as a function of condition m computed for the FEP/REST simulations in the DMPC bilayer (a) and lipid-free water (b). The maximum theoretical value h_o is marked by dashed line. The data is averaged across all FEP/REST trajectories.

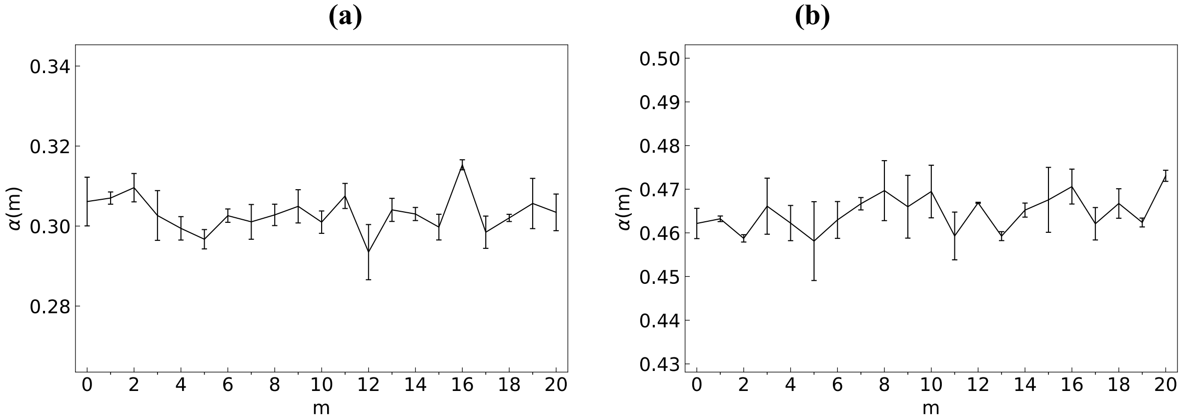

Figure S3 Replica exchange rates $\alpha(m)$ as a function of condition m computed for the FEP/REST simulations in the DMPC bilayer (a) and lipid-free water (b). The data is averaged across all FEP/REST trajectories.

Convergence of FEP/REST sampling: To evaluate the convergence of FEP/REST simulations, we computed the free energy changes occurring along the thermodynamic cycle in Fig. 2a, ΔG_{bl}(wt→ox) in the bilayer and ΔG_w(wt→ox) in lipid-free water, as a function of FEP/REST sampling time t. Specifically,

Fig. S4 tracks the variations in ΔG_{bl}(wt→ox) and ΔG_w(wt→ox) computed within the batches of 4 ns along FEP/REST sampling. Fig. S4a shows that ΔG_{bl}(wt→ox) approaches the baseline at $t > 276$ ns. Importantly, the equilibration is robust, because it is observed in ΔG_{bl}(wt→ox) computed for individual FEP/REST trajectories (see inset to Fig. S4a). Therefore, we assumed that the equilibration time in the FEP/REST simulations in the DMPC bilayer is t_{eq}=276 ns giving us that ΔG_{bl}(wt→ox) 24 ns of equilibrated sampling at each condition per trajectory. Fig. S4b reveals that ΔG_w(wt→ox) fluctuates around the baseline across the entire sampling range suggesting rapid equilibration. Accordingly, in lipid-free water FEP/REST collected 20 ns of equilibrated sampling per condition and trajectory. The total amount of equilibrated FEP/REST sampling is 1.512 µs in the bilayer and 1.26 µs in lipid-free water.

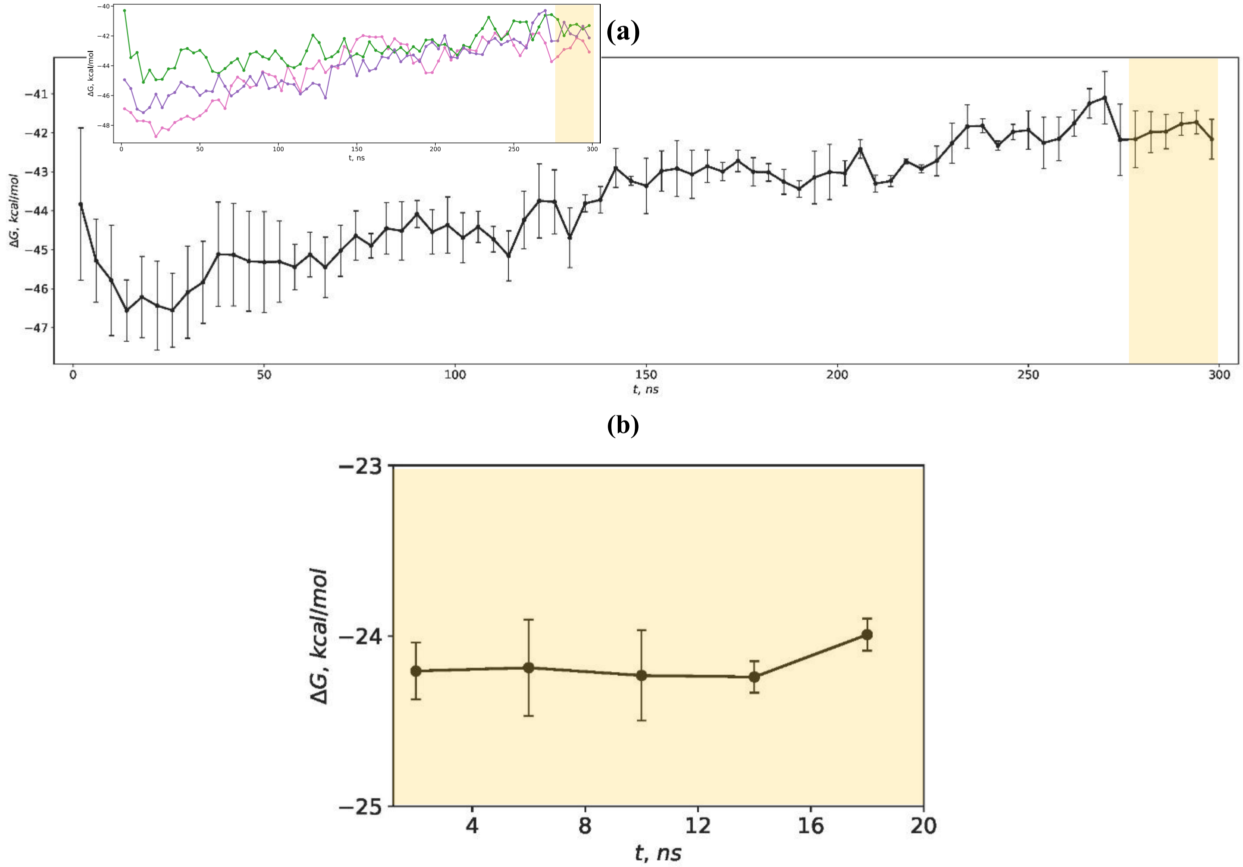

Figure S4 (a) The free energy change ΔG_{bl}(wt→ox) caused by wt→ox alchemical transformation in the DMPC bilayer computed as a function of FEP/REST sampling time t. The values of ΔG_{bl}(wt→ox) are computed using 4 ns sampling batches. The inset shows ΔG_{bl}(wt→ox) as a function of time t for three FEP/REST trajectories. Each trajectory is marked by different color. (b) Similar plot obtained for the FEP/REST simulations in lipid-free water. Shaded regions mark equilibrated sampling.

Mapping the heterogeneity of Aβ25-35 structures in the DMPC bilayer: To quantify the conformational ensemble of Aβ25-35 peptides in the DMPC bilayer, we used the alignment and clustering procedure outlined in Models and Methods. We first alligned all-vs-all Aβ25-35 structures by minimizing their respective RMSD. The probability distributions *P(RMSD)* for wtAβ25-35 and oxAβ25-35 are shown in Fig. S5. The distribution for wtAβ25-35 is bimodal suggesting the existance of distinct structural states. To map those, we performed clustering of wtAβ25-35 structures and discovered that in a wide range of RMSD cut-offs R_0 the structural ensemble partitions into two or three populated states. Their properties correspond

either to the states I or DI identified in Fig. 4a. We selected such value of R_0=7.0 Å that the two populated clusters, CL1 and CL2, perfectly "consume" the states I and DI. Indeed, at this R_0 98% of CL1 structures come from the state I, whereas 100% of CL2 structures originate from DI. Conversely, approximately 98% of the state I peptides belong to cluster CL1, and 97% of peptides from DI are present in CL2.

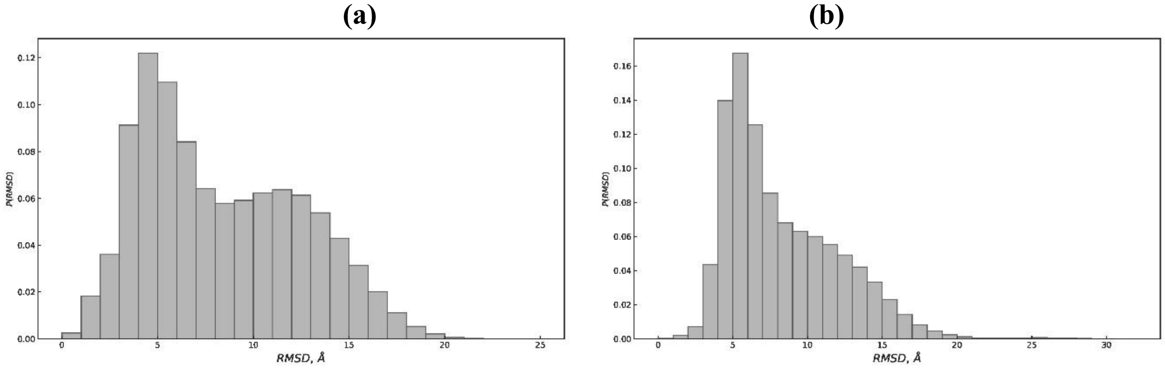

Figure S5 The probability distributions *P(RMSD)* of all-vs-all RMSD values computed for wtAβ25-35 (a) and oxAβ25-35 (b) peptides bound to the DMPC bilayer.

According to Fig. S5b existence of multiple populated clusters among oxAβ25-35 conformations is less apparent. However, applying the same procedure used for wtAβ25-35 we identified R_0=7.0 Å as the cut-off RMSD that maps perfectly two populated clusters onto the states I and SB observed in the probability distribution $P(z_{com})$ in Fig. 4b for oxAβ25-35. Specifically, 97% of CL1 structures come from the state SB, whereas 99% of CL2 structures originate from I. Conversely, approximately 96% of the state SB peptides belong to cluster CL1, and 86% of peptides from I occur in CL2.

Do wtAβ25-35 dimers change binding energetics or structural properties? In the main text we report that wtAβ25-35 aggregates within the DMPC bilayer with the probability of 0.15. To understand the consequences of transmembrane aggregation on binding, we analyzed the changes in free energy ΔG_{bl}(wt→ox) due to wt→ox alchemical transformation occurring when wtAβ25-35 aggregated states are excluded. The results are summarized in Table S3. The main conclusion is that transmembrane dimers have vanishingly small contribution to ΔG_{bl}(wt→ox) in Fig. 2a. Indeed, according to Table S3 ΔG_{bl}(wt→ox) does not change whether we include or exclude dimers from the computations. However, this outcome results from canceling two opposing factors. Dimeric states stabilize wtAβ25-35 bound state making an enthalpic gain for Aβ25-35 monomers slightly higher (ΔH_{bl} = -10.6 kcal/mol vs -9.6 kcal/mol in Table 1 in the main text). Additionally, the dimers have reduced conformational freedom resulting in weaker entropic gain for Aβ25-35 monomers ($T\Delta S_{bl}$ =10.4 kcal/mol vs 11.4 kcal/mol in Table 1). Nevertheless, these effects are subtle, because the respective changes in ΔH and $T\Delta S$ are ≈1 kcal/mol.

Table S3 Energetics of oxidation alchemical transformation in Aβ25-35 monomers in the DMPC bilayer.

**term**	value, kcal/mol
$\boldsymbol{\Delta G_{bl}}$	-21.0±0.2
$\boldsymbol{\Delta H_{bl}}$	-10.6±3.4
$\boldsymbol{T\Delta S_{bl}}$	10.4±3.4

We have also evaluated the impact of dimers on structural properties. The helical propensities *H* of monomeric wtAβ25-35 in the I and DI states are 0.50 and 0.85 compared to 0.50 and 0.82 if all peptide states are considered. Dimeric states also produce fairly minor impact on the probability distributions *P(z;i)* of amino acids along the bilayer normal or on the lipid density maps $n_l(r,z)$. For example, if wtAβ25-35

dimers are excluded, the bilayer thinning ΔD is 13.6±1.6 Å while it is 19.0±3.5 Å if all peptides are considered. Thus, we conclude that wtAβ25-35 transmembrane dimers do not skew our findings considerably.

Mapping binding interactions between amino acids and lipid groups: We computed the binding contact maps <$C_b(i,k)$> reporting the interactions between Aβ25-35 amino acids i and lipid groups k. Fig. S6 shows <$C_b(i,k)$> for wtAβ25-35 and oxAβ25-35. For both peptides the strongest binding interaction occurs between cationic Lys28 and phosphate group L2.

(a) **(b)**

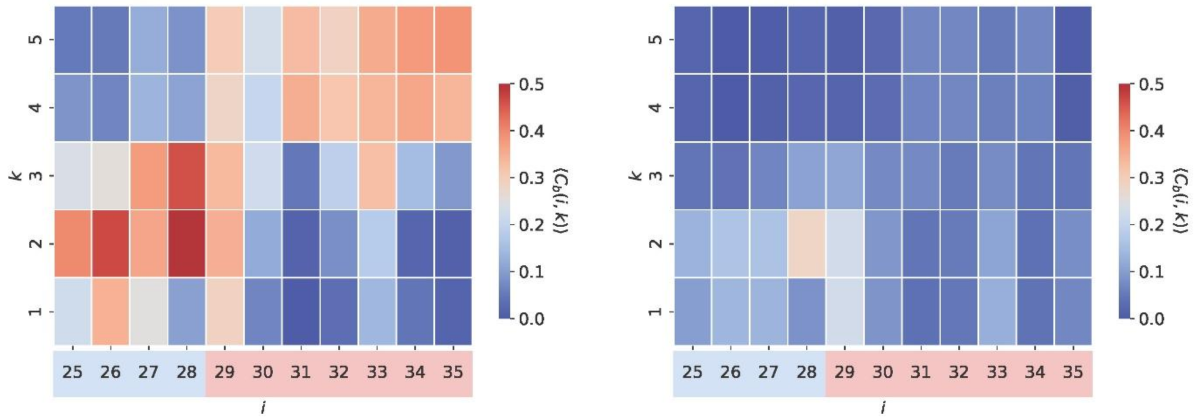

Figure S6 The binding contact maps <$C_b(i,k)$> report interactions between amino acids i and lipid groups k for wtAβ25-35 (a) and oxAβ25-35 (b).

References:

[1] D'Ursi, A. M., Armenante, M. R., Guerrini, R., Salvadori, S., Sorrentino, G., and Picone, D. (2004) Solution structure of amyloid β-peptide (25-35) in different media. *J. Med. Chem.* **47**, 4231-4238.

[2] Smith, A. K. and Klimov, D. K. (2018) Binding of cytotoxic Aβ25-35 peptide to the DMPC lipid bilayer. *J. Chem. Inform. Model.* **58**, 1053–1065.

[3] Khayat, E., Lockhart, C., Delfing, B.M., Smith, A. K., and Klimov, D. K. (2021) Met35 Oxidation Hinders Aβ25-35 Peptide Aggregation within the DMPC Bilayer. *ACS Chem. Neurosci.* **12**, 3225–3236.

[4] Laracuente, X. E., Delfing, B. M., Luo, X., Olson, A., Jeffries, W., Bowers, S. R., Foreman, K. W., Lee, K. H., Paige, M., Kehn-Hall, K., Lockhart, C., and Klimov, D. K. (2025) Applying absolute free energy perturbation molecular dynamics to diffusively binding ligands. *J. Chem. Theor. Comput.* **21**, 4286–4298.

[5] Han, M. & Hansmann, U. H. E. (2011) Replica exchange molecular dynamics of the thermodynamics of fibril growth of Alzheimer's Aβ42 peptide. *J. Chem. Phys.* **135**, 065101.

[6] Denschlag, R., Lingenheil, M., and Tavan, P. (2009) Optimal temperature ladders in replica exchange simulations. *Chem. Phys. Lett.* **473**, 193-195.
